# Increased genomic prediction accuracy in wheat breeding using a large Australian panel

**DOI:** 10.1007/s00122-017-2975-4

**Published:** 2017-09-08

**Authors:** Adam Norman, Julian Taylor, Emi Tanaka, Paul Telfer, James Edwards, Jean-Pierre Martinant, Haydn Kuchel

**Affiliations:** 1 School of Agriculture, Food and Wine, University of Adelaide, Waite Campus, Glen Osmond, SA Australia; 2 National Institute for Applied Statistics Research Australia (NIASRA), School of Mathematics and Applied Statistics, University of Wollongong, Wollongong, NSW Australia; 3 Australian Grain Technologies Pty Ltd, Perkins Building, Roseworthy Campus, Roseworthy, SA Australia; 4 Centre of Research, Limagrain Field Seeds Pty Ltd, Chappes, France

## Abstract

****Key message**:**

**Genomic prediction accuracy within a large panel was found to be substantially higher than that previously observed in smaller populations, and also higher than QTL-based prediction.**

**Abstract:**

In recent years, genomic selection for wheat breeding has been widely studied, but this has typically been restricted to population sizes under 1000 individuals. To assess its efficacy in germplasm representative of commercial breeding programmes, we used a panel of 10,375 Australian wheat breeding lines to investigate the accuracy of genomic prediction for grain yield, physical grain quality and other physiological traits. To achieve this, the complete panel was phenotyped in a dedicated field trial and genotyped using a custom Axiom^TM^ Affymetrix SNP array. A high-quality consensus map was also constructed, allowing the linkage disequilibrium present in the germplasm to be investigated. Using the complete SNP array, genomic prediction accuracies were found to be substantially higher than those previously observed in smaller populations and also more accurate compared to prediction approaches using a finite number of selected quantitative trait loci. Multi-trait genetic correlations were also assessed at an additive and residual genetic level, identifying a negative genetic correlation between grain yield and protein as well as a positive genetic correlation between grain size and test weight.

**Electronic supplementary material:**

The online version of this article (doi:10.1007/s00122-017-2975-4) contains supplementary material, which is available to authorized users.

## Introduction

Plant breeding has been successful in producing significant yield gains in wheat since the beginning of the twentieth century (Wrigley and Rathjen 1981); this has largely been driven by the innovation and adoption of new breeding technologies. Such progress is underpinned by extensive research, first in developing the technology, and second on establishing its application. If new technologies are to continue enabling plant breeding to deliver genetic gain to growers, innovative research must be undertaken in datasets that are relevant to the setting in which they will be applied.

Molecular markers are one technology that represent an invaluable research tool for understanding the genetic control of various traits. They have frequently been utilised in quantitative trait loci (QTL) mapping studies, and applied in breeding programmes through marker-assisted selection (MAS) (Koebner and Summers 2003; Collard and Mackill 2008). Early statistical modelling approaches to QTL mapping involved the analysis of individual markers through simple scanning procedures (Soller et al. 1976). In more modern approaches, statistical methods have improved the efficiency and power of QTL detection through the simultaneous incorporation of markers from the whole genome in complex linear mixed models (Zhang et al. 2010; Verbyla et al. 2012). There has also been focus on whole genome QTL mapping in broader multiparent populations (Huang et al. 2012; Sannemann et al. 2015; Mackay et al. 2014), and diverse association panels (Neumann et al. 2011; Bentley et al. 2014; Zanke et al. 2014). The latter usually involves the use of genome-wide association studies (GWAS) and has become a valuable tool for broad validation of previously identified QTL as well as identification of QTL in the target breeding germplasm. For qualitative traits under simple genetic control, GWAS, and subsequent application of MAS has been shown to be an effective tool in breeding programmes (Xu and Crouch 2008). However, for more complex polygenic quantitative traits such as grain yield, there have been few examples of genetic improvement using MAS (Dekkers et al. 2002). This deficiency can be overcome by implementing a genomic selection (GS) method that uses a complete set of molecular marker effects for predicting the performance of quantitative polygenic traits (Meuwissen et al. 2001). Current research in this area suggests with sufficient prediction accuracy, GS can be successfully applied in a breeding programme to increase rates of genetic gain (Cooper et al. 2014; Schmidt et al. 2016). Recent studies investigating the accuracy of GS in wheat have used population sizes ranging from several hundred to several thousand individuals, and achieved prediction accuracies mostly in the range of 0.50–0.60 as measured by Pearson correlation coefficients (Heslot et al. 2012; Nakaya and Isobe 2012; Isidro et al. 2015; He et al. 2016).

In GWAS and QTL analysis, the use of physical and genetic maps has been widely adopted (Kang et al. 2010; Zhang et al. 2010). Recombination information from these maps could also be used in GS programmes to simulate the progeny of specific parents for the purpose of designing crosses (Podlich and Cooper 1998). Physical maps are becoming available for wheat (Pozniak 2016), but can be of limited value if the individuals sequenced are not closely related to the target germplasm. Additionally, physical maps do not incorporate recombination information, which reduces their value when we are interested in simulating progeny based on recombination probabilities in the germplasm of interest. Therefore, high-quality genetic maps built from relevant germplasm are a better resource for these applications. Examples of such maps in the literature include those produced using multi-parent advanced generation inter-cross (MAGIC) populations (Huang et al. 2012; Gardner et al. 2016), as well as consensus maps constructed from multiple bi-parental populations (Cavanagh et al. 2013; Wang et al. 2014). These maps can also be used to measure the extent of linkage disequilibrium (LD) between markers (Zhao et al. 2005; Chao et al. 2010). In the context of association mapping and genomic prediction, LD becomes vitally important as it influences the achievable mapping resolution (Huang et al. 2012), power and accuracy of QTL detection (Somers et al. 2007), and the accuracy of genomic prediction in a breeding programme after multiple generations (Muir 2007). The extent of LD is also known to vary significantly depending on the germplasm structure (Hao et al. 2011; Huang et al. 2012) and as a consequence, assessments of LD should be conducted on the genetic material being studied.

For GS to be applied effectively, plant breeders must have a sound understanding of the relationship between traits of interest as it enables optimisation of selection strategies through correlated response to selection (Bernardo 2002). Trait correlations in bread wheat have long been reported at the phenotypic level (Bhatt and Derera 1975; Fischer and Wood 1979). Advances in statistical techniques have since made it possible to draw genetic correlations between traits by separating the genetic variance from the residual error (Gilmour et al. 1997), and these have been reported for various physiological traits in bread wheat (Rebetzke and Richards 1999; Sukumaran et al. 2015). These approaches, coupled with the use of pedigree or molecular marker information, can also be used to separate the genetic variance into its additive and residual components, thus allowing genetic correlations to be drawn at the additive and residual genetic level (Rebetzke et al. 2013). These genetic correlations, particularly the additive, provide a more precise measure of trait relationships and facilitate better optimisation of selection strategies.

In the present study we use a panel of 10,375 lines from a commercial wheat breeding programme to: (1) assess the level of LD using a constructed high-quality genetic consensus map; (2) investigate genetic correlations between traits at an additive and residual genetic level; (3) investigate the improvement in selection accuracy that is achieved by incorporating a genomic relationship matrix into the analysis model; (4) investigate the improvement in genomic prediction accuracy that is achievable with a germplasm of this size and compare it to a simplified prediction approach based on selection of finite QTL.

## Materials and methods

### Plant material and phenotype data

**Table 1:** Summary of the phenotype data and the methods used for collection

Trait	Assessment method	Scale	Mean	SD
Growth habit	Visual	1–9; 1 $=$ erect	2.4	1.0
Leaf width	Visual	1–9; 1 $=$ narrow	4.8	1.4
Biomass	Visual	1–9; 1 $=$ low biomass	6.9	1.3
NDVI	GreenSeeker$^{\mathrm{a}}$	NDVI	0.68	0.1
Physiological yellows	Visual	1–9; 1 $=$ low expression	1.7	0.9
Relative maturity	Visual	Zadoks scale$^{\mathrm{b}}$	53	5.7
Greenness	Visual	1–9; 1 $=$ pale green	5.7	1.5
Glaucousness	Visual	1–9; 1 $=$ low expression	3.5	2.0
Leaf loss	Visual	1–9; 1 $=$ low loss	4.6	1.7
Plant height	Visual	1–9; 1 $=$ short	5.2	1.1
Grain yield	Machine harvester	kg/ha	5124	655
Test weight	Chondrometer	kg/hl	84.4	1.8
Thousand kernel weight	Image analysis	TKW	37.5	4.6
Grain protein	NIR$^{\mathrm{c}}$	Concentration (%)	11.1	0.9

Mean and standard deviation are calculated from the raw phenotype data

$$^{\mathrm{a}}$$ Trimble (2016)

$^{\mathrm{b}}$ Zadoks et al. (1974)

$$^{\mathrm{c}}$$ Zeutec (2016)

A panel of diverse bread wheat lines was provided by Australian Grain Technologies Pty Ltd (AGT). The panel consists of lines from preliminary yield testing (PYT) and advanced yield testing (AYT) stages of the AGT breeding programmes. Online Resource 1 summarises the panel and its subsets. The PYT-South and AYT-South sets are comprised of lines bred for southern Australia, and the AYT-Other set represents lines from the north eastern, eastern, and western growing regions. PYT material is a combination of F$_{2}$ and F$_{5}$ derived lines, whereas AYT lines are derived from the F$_{5}$ generation or later. By including germplasm from both preliminary and advanced stages of the breeding programme, a set of unselected lines exist for each trait of interest. The panel was phenotyped in 2014 in a dedicated field trial at Roseworthy, South Australia (−34.52, 138.69), which was sown as a non-replicated randomised design with repeated grid checks (1 check per 11 plots). The trial was non-replicated as the large number of lines in the AWP made loading a replicated trial logistically infeasible. Dimensions of the trial were 476 rows by 24 ranges, and plot size was 3m$^{2}$. The trial was managed according to best local practice including fertiliser applications to maximise grain yield and grain quality, and fungicide applications to control disease. Table 1 details the phenotyping methods and summarises the data for each trait, while Online Resource 2 highlights the phenotypic differences between the germplasm sets. Raw phenotype data are provided in Online Resource 3.

### Genotype data

#### Genotyping platform

Marker genotyping was performed using a custom Axiom^TM^ Affymetrix array containing 18,101 SNP markers. To build the customised array, SNPs were selected from previous variant identifications and SNP screenings in a range of genotyping platforms. The most prominent platform was a high-density Axiom^TM^ array developed in the collaborative French BreedWheat project (Etienne Paux, personal communication) consisting of 420,000 diverse SNPs. This was used to genotype a panel of approximately 200 wheat accessions from a range of geographic regions (western Europe, eastern Europe, North America, Australia, and exotic sources) for use in SNP selection. To achieve adequate and even coverage of the genome, SNPs were clustered into 20,000 groups based on a linkage disequilibrium threshold of $r^{2} = 0.96$. One SNP per group was then selected based on technical quality, information content, and to have a call rate greater than 70%. It was ensured that SNPs could be accurately read as co-dominant markers by confirming they generated clear allele clusters, and required fewer probes. A final selection was then carried out based on initial batches from the 20K array, and 18,101 of the most reliable and reproducible SNPs were selected. This final selection of SNPs was used to build the custom 18K Axiom^TM^ 384 layout array from Affymetrix. Arrays were read using the GeneTitan Multi-Channel Instrument, and allele calls were made using Axiom^TM^ Analysis Suite software by Affymetrix.

#### Consensus map

To provide an accurate assessment of LD between SNP markers in the AWP a consensus map was constructed using nine doubled haploid (DH) populations (Online Resource 1) genotyped on the custom Axiom^TM^ Affymetrix array. The DH populations represent key families of Australian wheat germplasm and were chosen to maximise polymorphic markers across the genome. The individual SNP DH linkage maps were constructed using a synergistic combination of the R/qtl (Broman and Sen 2009; Broman and Wu 2015) and R/ASMap (Taylor and Butler 2017) packages available in the R statistical computing environment (R Development Core Team 2015). Before construction, individual marker sets were thoroughly diagnostically checked and problematic lines and markers containing excessive segregation distortion or missing values were removed. For each DH population, markers were clustered and optimally ordered using the MSTmap (Wu et al. 2008) functionality available in R/ASMap. The individual constructed linkage maps were scrutinized and lines with excessive recombination or markers exhibiting large numbers of double crossovers removed. Chromosomal alignment of linkage maps occurred sequentially with initial alignment of the Kukri/RAC875 SNP map performed using legacy markers from the pre-existing Kukri/RAC875 SSR/DArT map (Bennett et al. 2012; Edwards 2012). All other DH SNP linkage maps were then aligned to the Kukri/RAC875 SNP map through commonality of markers. A summary of the final individual DH linkage maps and their common markers is given in Online Resource 4.

The complete set of nine DH linkage maps (marker names and positions) were then used in MergeMap (Wu et al. 2011) to form a consensus map. To ensure the greatest marker position accuracy, the population size for each bi-parental linkage map was also passed to MergeMap as a set of pre-defined weights. A total 13,747 markers were assigned to linkage groups and relative positions across the 21 chromosomes of the wheat genome. The MergeMap algorithm is known to inflate consensus map linkage group distances (Close et al. 2009; Cavanagh et al. 2013; Wang et al. 2014). Scaling of the consensus map in this research used a minimum mean square criterion. Let $M_{ijk}$ be the position of the *k*th marker in the *j*th linkage group of the *i*th bi-parental linkage map and $C_{jk}$ be the position of the equivalent marker in the *j*th linkage group of the consensus map. The optimal scaling factor $R_j$ applied to the *j*th consensus linkage group was then derived using

$$\begin{aligned} {{\mathrm{arg\,min}}}_{R_j \in {\mathbb {R}}} \sum _{i = 1}^9N_{ij}\sum _{k = 1}^{N_{i}}(C_{jk}R_j - M_{ijk})^2 \end{aligned}$$

The function is easily minimised by considering $R_j= \bar{D}_j/D_j^c$ where $D_j^c$ is the length of the *j*th observed consensus linkage group and profiling $\bar{D}_j$ over a conservative window in the vicinity of the average length of *j*th linkage groups from the bi-parental linkage maps. This procedure was repeated for all 21 chromosomes and the consensus map was scaled accordingly. Assessment of LD was then based on these scaled positions within each of the chromosomes. Table 2 summarises the consensus map by detailing individual chromosomes, chromosome groups and genomes, while final scaled (as well as unscaled) consensus map positions for the 13,747 markers are given in Online Resource 4.

**Table 2:** Summary of the consensus linkage map

	Total markers	Map positions	Markers per map position	Genetic length	Mean interval^a^
1A	838	308	2.7	129	0.42
1B	905	250	3.6	136	0.55
1D	222	112	2.0	137	1.22
2A	777	226	3.4	128	0.57
2B	1074	286	3.8	147	0.51
2D	204	109	1.9	159	1.46
3A	909	267	3.4	156	0.58
3B	1175	282	4.2	145	0.51
3D	246	120	2.1	152	1.27
4A	652	276	2.4	168	0.61
4B	490	184	2.7	113	0.61
4D	237	120	2.0	129	1.08
5A	922	350	2.6	190	0.54
5B	1057	340	3.1	172	0.51
5D	236	147	1.6	198	1.35
6A	590	208	2.8	127	0.61
6B	893	237	3.8	114	0.48
6D	209	101	2.1	142	1.40
7A	1068	319	3.3	164	0.51
7B	814	221	3.7	147	0.66
7D	229	140	1.6	171	1.22
Genome A	5756	1954	2.9	1062	0.54
Genome B	6408	1800	3.6	974	0.54
Genome D	1583	849	1.9	1088	1.28
Group 1	1965	670	2.9	403	0.60
Group 2	2055	621	3.3	434	0.70
Group 3	2330	669	3.5	453	0.68
Group 4	1379	580	2.4	410	0.71
Group 5	2215	837	2.6	560	0.67
Group 6	1692	546	3.1	383	0.70
Group 7	2111	680	3.1	482	0.71
Total	13,747	4603	3.0	3124	0.68

$^{\mathrm{a}}$ Mean interval (cM) between unique map positions

#### Imputation

Before imputation, markers were omitted if they had a minor allele frequency less than 1%. The remaining markers in the SNP array had a low missing call rate of 1%. The substantial numerical dimensions of the complete SNP array made it computationally impractical to impute missing allele scores using algorithms based on unclustered and unsorted markers (Rutkoski et al. 2013). To reduce this computational burden, chromosomal identifications of the markers from the consensus map were used to subset the SNP marker set. The remaining 4354 markers with no consensus map chromosomal assignment were then linked to these subsets using LD. For each chromosome subset, the K-nearest neighbour (KNN) method (Troyanskaya et al. 2001) implemented in the R package pedicure (Butler 2016) was used to impute missing allele calls from the weighted average of the data points at the nearest 10 markers. The complete marker matrix of 10,375 lines by 17,181 markers from herein was defined as $\mathbf {M}$.

### Statistical methods

#### Statistical modelling

An initial baseline linear mixed model was used to provide a preliminary assessment of the genetic variation of the traits collected from the Roseworthy trial. For a given vector of trait observations, $\mathbf {y} = (y_1, \ldots , y_n)$, the linear mixed model had the form

1 $$\begin{aligned} {\mathbf {y}} = {\mathbf {X}\mathbf {\tau }} + {\mathbf {Z}\mathbf {u}} + {\mathbf {Z}}_g{\mathbf {g}}_t + {\mathbf {e}} \end{aligned}$$

Here, $\mathbf {\tau }$ is a vector of fixed effects, with associated design matrix $\mathbf {X}$, and contained an intercept and potential coefficients for covariates in $\mathbf {X}$ explaining trends across the experimental layout. Non-genetic variation associated with the design of the experiment, such as blocks in the experimental area, was accounted for through the random effects $\mathbf {u}$ with indicator design matrix ${\mathbf {Z}}$ with ${\mathbf {u}} \sim N({\mathbf {0}}, \sigma ^2_u{\mathbf {I}})$. Other remaining sources of non-genetic environmental variation were modelled through the residual error $\mathbf {e}$ which was assumed to have the form ${\mathbf {e}} \sim N({\mathbf {0}}, \sigma ^2{\mathbf {R}})$ with ${\mathbf {R}} = {\mathbf {\Sigma }}_r(\rho _r) \otimes {\mathbf {\Sigma }}_c(\rho _c)$ defining a two-dimensional separable AR1 $\otimes$ AR1 correlation structure in the rows and column direction of the experiment (Gilmour et al. 1997). In the baseline model the total genetic variation of the 10,375 AWP lines was captured using the random effects ${\mathbf {g}}_t$ with indicator design matrix ${\mathbf {Z}}_g$ which maps AWP lines to the appropriate random effects in ${\mathbf {g}}_t$. These effects were assumed to have the distribution ${\mathbf {g}}_t \sim N(\mathbf {0}, \sigma ^2_t{\mathbf {I}})$ and the set of effects $({\mathbf {u}}, {\mathbf {g}}_t, {\mathbf {e}})$ were considered to be mutually independent.

For each of the traits, the baseline model (1) was then extended by partitioning the total genetic effects into additive marker and residual genetic effects to form the marker linear mixed model

2 $$\begin{aligned} {\mathbf {y}}&= {\mathbf {X}\mathbf {\tau }} + {\mathbf {Z}\mathbf {u}} + {\mathbf {Z}}_g({\mathbf {M}}{\mathbf {g}}_m + {\mathbf {g}}_p) + {\mathbf {e}} \end{aligned}$$

where ${\mathbf {g}}_m$ is a vector of marker effects and ${\mathbf {g}}_p$ is a vector of residual genetic effects. The effects were assumed to be distributed ${\mathbf {g}}_m \sim N({\mathbf {0}}, \sigma ^2_a{\mathbf {I}})$ and ${\mathbf {g}}_p \sim N({\mathbf {0}}, \sigma ^2_p{\mathbf {I}})$ with $({\mathbf {u}}, {\mathbf {g}}_m, {\mathbf {g}}_p, {\mathbf {e}})$ mutually independent. The large number of markers in $\mathbf {M}$, coupled with the substantial number of lines in the population made the fitting of (2) computationally prohibitive. For this reason an alternative formulation using the approach of Strandén and Garrick (2009) was sought. Let ${\mathbf {g}}_a$ define a set of additive genotype effects with ${\mathbf {g}}_a = {\mathbf {M}\mathbf {g}}_m$ then the genotype linear mixed model used was

3 $$\begin{aligned} {\mathbf {y}} = {\mathbf {X}\mathbf {\tau }} + {\mathbf {Z}\mathbf {u}} + {\mathbf {Z}}_g({\mathbf {g}}_a + {\mathbf {g}}_p) + \mathbf {e} \end{aligned}$$

where $\mathbf {g}_a \sim N({\mathbf {0}}, \sigma ^2_a{\mathbf {G}})$ and ${\mathbf {G}} = {\mathbf {M}\mathbf {M}}^T$ is a $10,375 \times 10,375$ additive relationship matrix. For the purpose of providing an appropriate scaling, $\mathbf {G}$ was replaced by ${\mathbf {G}}_s = {\mathbf {M}}{\mathbf {M}}^T/r$ with $r = {\text{ trace }}({\mathbf {G}})/10{,}375$ (Forni et al. 2011). An eigen decomposition of ${\mathbf {G}}_s$ revealed only positive eigenvalues indicating ${\mathbf {G}}_s$ was positive definite and could be safely inverted.

Estimation of the parameters for the linear mixed models (1) and (3) occurred iteratively. Fixed effect estimates and predictions of random effects were determined through direct solving of the mixed model equations (Henderson 1953). Variance parameters were estimated using residual maximum likelihoood (REML) (Patterson and Thompson 1971). From the fitted baseline model (1) broad sense heritabilities were then calculated for each of the traits using REML estimates of the variance parameters, namely $H^2 = \sigma ^2_t/(\sigma ^2_t + \sigma ^2)$. For the fitted additive genotype model (3) the broad sense heritability was calculated by replacing the total genetic variability in $H^2$ by $\sigma _t^2 = \sigma _a^2 + \sigma _p^2$. Narrow sense heritabilities were also calculated using $h^2 = \sigma ^2_a/(\sigma ^2_t + \sigma ^2)$.

#### Genomic prediction

Using mixed model results, genomic best linear unbiased predictions of the additive genetic effects $\mathbf {g}_a$ and predictions of the residual genetic effects ${\mathbf {g}}_p$ in (3) were immediately determined for each trait using

4 $$\begin{aligned} \tilde{\mathbf {g}}_a&= \sigma ^2_a{\mathbf {G}}_s{\mathbf {Z}}_g^T{\mathbf {P}}{\mathbf {y}}\nonumber \\ \tilde{\mathbf {g}}_p&= \sigma ^2_p{\mathbf {Z}}_g^T{\mathbf {P}}{\mathbf {y}} \end{aligned}$$

where ${\mathbf {P}} = {\mathbf {H}}^{-1} - {\mathbf {H}}^{-1}{\mathbf {X}}({\mathbf {X}}^T{\mathbf {H}}^{-1}{\mathbf {X}})^{-1}{\mathbf {X}})^{-1}{\mathbf {X}}^T{\mathbf {H}}^{-1}$ and ${\mathbf {H}} = \sigma ^2{\mathbf {R}} + \sigma ^2_u{\mathbf {Z}}{\mathbf {Z}}^T + {\mathbf {Z}}_g(\sigma ^2_a{\mathbf {G}}_s + \sigma ^2_p{\mathbf {I}}){\mathbf {Z}}_g^T$. The additive genetic effects, $\tilde{\mathbf {g}}_a$ reflect the breeding value of lines estimated from phenotpyic and genetic information. Both $\tilde{\mathbf {g}}_a$ and $\tilde{\mathbf {g}}_p$ were used to investigate the additive and residual genetic relationships between the analysed Roseworthy traits.

From the marker linear mixed model (2), predicted marker effects were immediately calculated using

5 $$\begin{aligned} \tilde{\mathbf {g}}_m = \sigma ^2_a{\mathbf {M}}^T{\mathbf {Z}}_g^T{\mathbf {P}}{\mathbf {y}} = \mathbf {M}^T{\mathbf {G}}_s^{-1}\tilde{\mathbf {g}}_a \end{aligned}$$

This result ensured the marker effects were efficiently derived from the additive genetic values for the lines given by (4). Inversion of ${\mathbf {G}}_s$ would usually be computationally expensive but was very efficient using the highly parallelised Basic Linear Algebra Subprograms available in the Intel^TM^ Math Kernel Libraries. Given a new set of lines with marker data ${\mathbf {M}}^*$ genotyped across identical markers in $\mathbf {M}$, genomic predictions for the new lines can then be determined using the simple equation $\tilde{\mathbf {g}}^* = \mathbf {M}^*\tilde{\mathbf {g}}_m$, utilizing the complete set of predicted marker effects.

To evaluate the power of the genomic prediction approach using the results derived from the full additive genotype linear mixed model (3), it was compared to a simplified prediction approach based on finite selection of putative QTL. To provide a mechanism for selecting important markers linked to a QTL for each of the traits, the complete set of marker outlier statistics were calculated using the formula derived in Verbyla et al. (2007). For any given trait, the *k*th marker outlier statistic is

$$\begin{aligned} t_k = \frac{\tilde{g}_{m;k}^2}{\text{ var }(\tilde{g}_{m;k})} \end{aligned}$$

where $\tilde{g}_{m;k}$ is the *k*th marker effect obtained directly from (5) with its variance extracted from the diagonal components of the variance matrix ${\text{ var }}(\tilde{\mathbf {g}}_m) = {\mathbf {M}}^T{\mathbf {G}}_s^{-1}\text{ var }(\tilde{\mathbf {g}}_a){\mathbf {G}}_s^{-1}{\mathbf {M}}$. In most modern linear mixed modelling software ${\text{ var }}(\tilde{\mathbf {g}}_a)$ is usually available from the fitted additive genotype model in (3), ensuring efficient computing of the variance of the predicted marker effects. For each of the traits, the largest one and five marker outlier statistics were identified iteratively using a consensus map exclusion window of 25cM either side of any selected marker. The selected markers were then extracted from $\mathbf {M}$, denoted ${\mathbf {M}}_1$ and $\mathbf {M}_5$, respectively, placed in the baseline model (1) as an additive set of QTL fixed effects

6 $$\begin{aligned} \mathbf {y} = \mathbf {Z}_g\mathbf {M}_j\mathbf {\beta }_j + \mathbf {X}\mathbf {\tau } + \mathbf {Z}\mathbf {u} + \mathbf {Z}_g\mathbf {g}_p + \mathbf {e} \end{aligned}$$

where $j = (1, 5)$ and ${\mathbf {\beta }}_j$ are the QTL fixed effect parameters for the selected markers in ${\mathbf {M}}_j$. In this model, ${\mathbf {g}}_t$ has been replaced with a residual genetic effect ${\mathbf {g}}_p$ as the inclusion of markers strongly linked to QTL will absorb genetic variation. The genetic value of the lines were then calculated directly from the equation $\tilde{\mathbf {g}}_a = {\mathbf {M}}_j\hat{\mathbf {\beta }}_j$, where $\hat{\mathbf {\beta }}_j$ are estimates of the QTL fixed effects extracted from the fitted model of (6). Similarly, given a new set of lines with marker data for the selected markers, $\mathbf {M}_j^*$, QTL-based predictions for the new lines can be calculated using $\tilde{\mathbf {g}}^* = {\mathbf {M}}_j^*\hat{\mathbf {\beta }}_j$.

#### Prediction accuracy

To provide an informative comparison with genomic prediction results discussed in the plant research literature, the predictive ability of the fitted additive genotype model (3), as well as of predictions obtained using selected QTL effects estimated from the fitted model of (6), was calculated for each of the traits using fivefold cross-validation. The cross-validation method initially randomly partitioned the AWP lines into five equal subsets. Let $({\mathbf {g}}_a^{(i)}, {\mathbf {g}}_p^{(i)})$ be the additive and residual genetic effects of the AWP lines in the *i*th subset (validation set) and $({\mathbf {g}}_a^{(-i)}, {\mathbf {g}}_p^{(-i)})$ the additive and residual genetic effects of the AWP lines remaining in the other four (training set). The cross-validation for each prediction method was conducted sequentially for each of the folds $i = 1,\ldots ,5$. For the genomic prediction approach incorporating the additive relationship matrix, $({\mathbf {g}}_a^{(-i)}, {\mathbf {g}}_p^{(-i)})$ were fitted as additive and residual genetic effects in the additive genotype model, the additive genetic values for $\tilde{\mathbf {g}}_a^{(-i)}$ were derived using (3) and marker effects, $\tilde{\mathbf {g}}_m^{(-i)}$, were calculated using (5). The AWP lines in the *i*th validation set were then predicted using $\tilde{\mathbf {g}}_a^{(i)} = \mathbf {M}_j^{(i)}\tilde{\mathbf {g}}_m^{(-i)}$. For prediction methods using selected QTL, ${\mathbf {g}}_p^{(-i)}$ was fitted in (6) and QTL effects $\hat{\mathbf {\beta }}_j^{(-i)}$ were extracted and used to calculate predictions for the validation set of AWP lines using $\tilde{\mathbf {g}}_a^{(i)} =\mathbf {M}_j^{(i)}\hat{\mathbf {\beta }}_j^{(-i)}$. Prediction accuracies were calculated by correlating the validation set predictions obtained from each cross-validation fold, $\{\tilde{\mathbf {g}}_a^{(i)}; i = 1,\ldots ,5\}$, to their full additive genetic values $(\tilde{\mathbf {g}}_a)$ extracted from the additive genotype model containing the complete set of lines. To enable the comparison of these results to those of previous studies, validation set predictions were also correlated to their corresponding total genetic values obtained from the baseline model, and divided by the square root of the heritability of the baseline model (Heffner et al. 2011b; Estaghvirou et al. 2013; Battenfield et al. 2016). Comparing predictions to both total and additive genetic values enabled an assessment of prediction accuracy to be made for line selection and parental value, respectively.

#### Computations

All linear mixed modelling was conducted using the ASReml-R package (Butler et al. 2009) available in the R statistical computing environment (R Core Team 2017). Trait models containing the full additive relationship matrix took an average of 60 h computational time to converge on a Windows 10 box with a quad core Intel^TM^ i7-6700K (4.00Ghz) with 64Gb RAM.

## Results

### Linkage disequilibrium

**Fig. 1:**
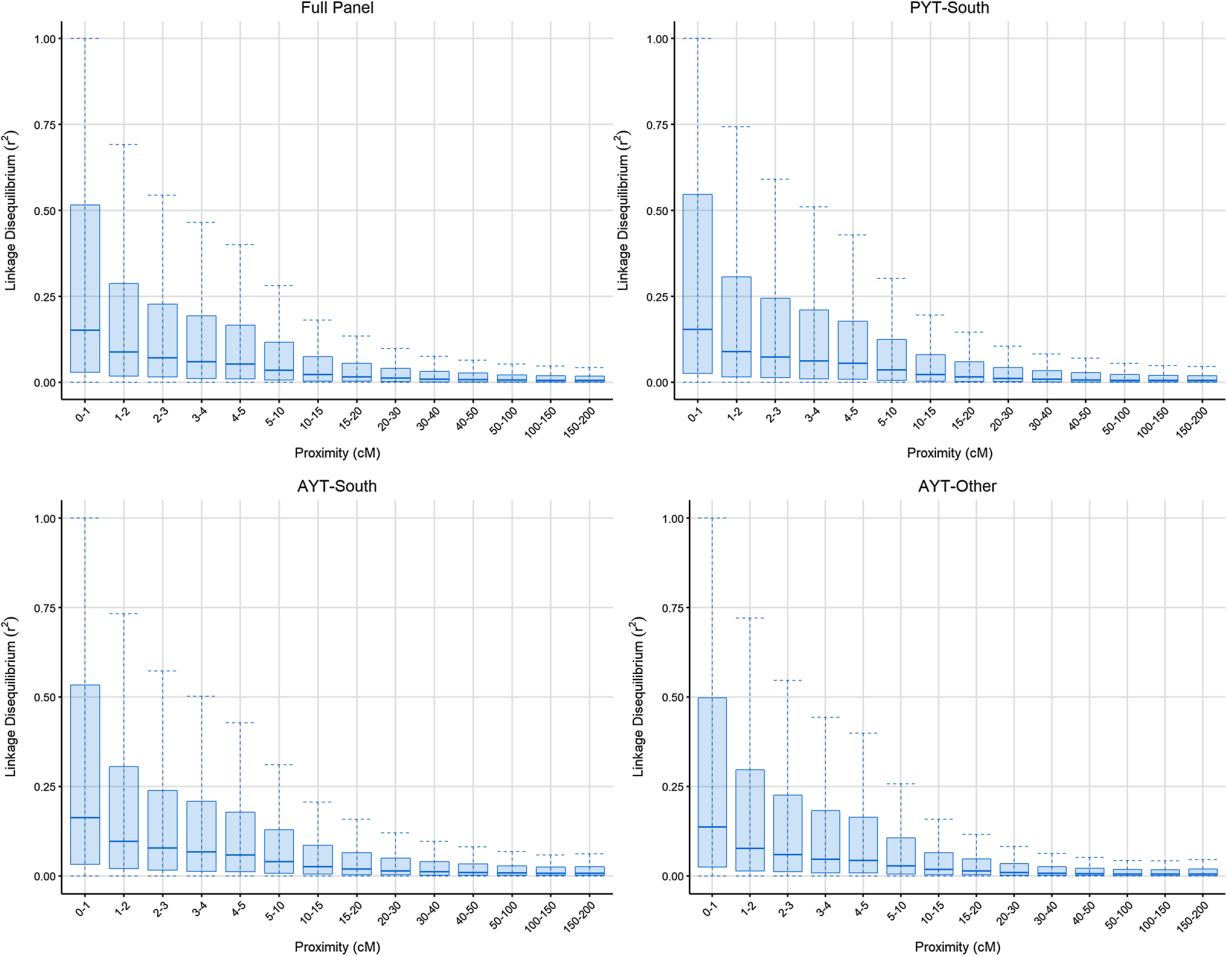
Boxplots comparing linkage disequilibrium $(r^2)$ of marker pairs with their proximity on the consensus map

Linkage disequilibrium was assessed by calculating $r^{2}$ values between marker pairs within each consensus map chromosome (Fig. 1). In the full panel, the median $r^{2}$ for marker pairs with proximity less than 2 cM is just 0.12, and this steadily decreases as the distance between a pair of markers increases. However, there is significant variation in the $r^{2}$ value between markers in very close proximity, with some being in complete LD with each other. The boxplots clearly show that this variation decreases rapidly with increasing distance, and plateaus off after proximity exceeds 20 cM. The broad pattern of LD decay was very similar for each of the germplasm sets, but there were subtle differences for close marker pairs (${<}2$ cM) with AYT-South showing slightly higher LD than PYT-South, which itself was higher than AYT-Other.

### Genetic trait correlations

**Table 3:** Pairwise genetic correlations between traits from the Roseworthy experiment

	Bm.	Gl.	GP	GY	Gr.	GH	PH	LL	LW	Mat.	NDVI	TW	TKW	Yl.
Biomass	–	$-0.24$	$-0.39$	0.49	$-0.44$	$-0.45$	0.10	0.69	0.49	0.76	0.51	0.15	0.19	$-0.34$
Glaucousness	$-0.18$	–	0.41	$-0.01$	0.73	$-0.23$	$-0.04$	$-0.41$	0.24	$-0.30$	$-0.28$	$-0.04$	0.13	0.41
Grain protein	$-0.14$	0.15	–	$-0.55$	0.50	0.10	0.02	$-0.40$	$-0.08$	$-0.39$	$-0.34$	$-0.22$	$-0.23$	0.35
Grain yield	0.27	$-0.03$	$-0.30$	–	0.06	$-0.06$	0.11	0.01	0.10	0.19	0.16	0.28	0.23	$-0.19$
Greenness	$-0.14$	0.23	0.22	$-0.15$	–	$-0.14$	$-0.05$	$-0.61$	0.02	$-0.43$	$-0.45$	$-0.02$	$-0.01$	0.49
Growth habit	$-0.15$	0.07	0.20	$-0.30$	0.11	–	0.15	$-0.36$	$-0.59$	$-0.48$	0.25	$-0.10$	$-0.30$	$-0.25$
Plant height	0.19	$-0.14$	$-0.04$	$-0.27$	$-0.08$	0.05	–	$-0.10$	$-0.01$	$-0.10$	0.12	0.09	$-0.04$	$-0.16$
Leaf loss	0.32	$-0.16$	$-0.22$	0.28	$-0.26$	$-0.23$	$-0.05$	–	0.33	0.75	0.31	0.10	0.23	$-0.18$
Leaf width	0.22	$-0.04$	$-0.05$	0.30	$-0.03$	$-0.11$	0.06	0.13	–	0.43	0.04	0.05	0.37	0.09
Maturity	0.42	$-0.19$	$-0.30$	0.36	$-0.19$	$-0.32$	0.01	0.45	0.19	–	0.24	0.24	0.29	$-0.20$
NDVI	0.34	$-0.13$	$-0.01$	0.43	$-0.10$	0.12	0.20	0.14	0.05	0.08	–	0.04	0.06	$-0.51$
Test weight	0.12	$-0.10$	$-0.43$	0.29	$-0.09$	$-0.17$	0.01	0.02	0.02	0.21	0.12	–	0.37	0.00
TKW	0.14	$-0.08$	$-0.33$	0.39	$-0.08$	$-0.20$	0.06	0.12	0.15	0.35	$-0.06$	0.52	–	0.11
Yellows	$-0.11$	0.04	0.00	$-0.25$	$-0.04$	$-0.07$	$-0.15$	0.06	$-0.01$	$-0.06$	$-0.04$	$-0.01$	$-0.05$	–

Additive genetic correlations are in the upper triangle and residual genetic are in the lower triangle

From each of the traits, the additive genetic values and residual genetic values were extracted from their respective fitted additive genotype models and used to understand genetic relationships between the traits. Table 3 presents the pairwise additive and residual genetic correlations between traits analysed in the 2014 Roseworthy field trial. The two correlation measures largely agreed, with a correlation of 0.79 across the 91 trait pairs. Of the 91 trait pairs, 74 had correlations in the same direction, and those that differed in direction were all near zero. Additive genetic correlations were overall stronger than residual genetic with an absolute mean of 0.26 compared to 0.14. Notable correlations include the well-known strong negative relationship between grain yield and grain protein, with an additive correlation of −0.55 and a residual genetic of −0.30. A negative relationship was also observed between grain protein and test weight (additive correlation −0.22, residual genetic −0.43). Strong positive relationships were observed between test weight and thousand kernel weight (TKW) (additive correlation: 0.37, residual genetic 0.52), and relative maturity score and biomass (additive correlation 0.76, residual genetic 0.42).

### A comparison of additive and baseline models

**Table 4:** Comparison of the baseline and genomic mixed linear models

	Baseline model		Genomic model			
	*H* ^2^	Log* l*	*H* ^2^	*h* ^2^	Log* l*	Add. var. (%)^a^
Biomass	0.56	$-4113$	0.75	0.56	$-2401$	75
Glaucousness	0.81	$-12{,}424$	0.89	0.76	$-8370$	86
Grain protein	0.57	$-1119$	0.75	0.62	1517	82
Grain yield	0.44	$-76{,}861$	0.63	0.45	$-75{,}322$	72
Greenness	0.64	$-9271$	0.75	0.58	$-6479$	77
Growth habit	0.71	$-4148$	0.89	0.78	$-1781$	88
Plant height	0.74	$-5212$	0.91	0.81	$-2655$	89
Leaf loss	0.67	$-10{,}067$	0.83	0.69	$-7648$	82
Leaf width	0.71	$-7888$	0.86	0.75	$-4674$	87
Maturity	0.92	$-24{,}045$	0.98	0.91	$-20{,}562$	93
NDVI	0.45	25, 269	0.62	0.36	26, 160	58
Test weight	0.75	$-10{,}566$	0.91	0.82	$-7546$	90
TKW	0.79	$-21{,}047$	0.93	0.85	$-17076$	91
Yellows	0.73	$-3662$	0.82	0.53	$-2418$	65

Broad sense heritabilities are presented for each model, and narrow sense for the genomic model as there is no term in the base model to capture the additive genetic variance. Model fit is compared through the log-likelihood measure

^a^ Proportion of the variance accounted for by the model that is additive

All traits collected from the Roseworthy experiment were analysed and results from the fitted baseline models and additive genotype linear mixed models are compared in Table 4. Additive models had significantly higher log-likelihood (model fit) for all traits, with an average improvement of 44% over the equivalent baseline models. The additive model also improved broad sense heritability for all traits, with an average increase of 24%. Narrow sense heritabilities of the additive models were comparable with the broad sense heritability from the equivalent baseline models, being just 0.5% lower on average. The proportion of the genetic variance that was additive averaged 81% across all traits, and ranged from 58% (NDVI) to 91% (grain size). There was a strong positive relationship between the improvement in model fit obtained with the additive model and narrow sense heritability ($r = 0.86$).

### Prediction accuracy

**Table 5:** Fivefold cross-validation accuracy of genomic and QTL prediction models (one and five QTL)

	Genomic		One QTL		Five QTL	
	Additive ^a^	Total ^b^	Additive	Total	Additive	Total
Biomass	0.97	0.72	0.26	0.20	0.46	0.48
Glaucousness	0.98	0.82	0.49	0.45	0.76	0.68
Grain protein	0.97	0.84	0.16	0.16	0.59	0.54
Grain yield	0.97	0.71	0.19	0.16	0.64	0.51
Greenness	0.98	0.80	0.54	0.44	0.78	0.65
Growth habit	0.96	0.75	0.36	0.30	0.59	0.50
Plant height	0.96	0.76	0.28	0.24	0.48	0.43
Leaf loss	0.97	0.77	0.41	0.37	0.55	0.54
Leaf width	0.98	0.81	0.26	0.24	0.54	0.46
Maturity	0.96	0.77	0.26	0.25	0.59	0.55
NDVI	0.96	0.56	0.20	0.15	0.42	0.31
Test weight	0.96	0.80	0.10	0.11	0.43	0.39
TKW	0.97	0.85	0.38	0.33	0.52	0.49
Yellows	0.97	0.55	0.17	0.15	0.63	0.41

^a^ Correlation between the predicted values and the additive genetic values from the full genomic model

^b^ Correlation between the predicted values and the total genetic values from the baseline model, divided by the square root of the broad sense heritability

Table 5 presents the fivefold cross-validation accuracies of the genomic predictions and QTL-based predictions for all 14 traits. Prediction accuracy was assessed by correlating genomic and QTL-based predictions to both the additive genetic values from the full additive genotype model (shown to be the model of best fit for every trait, Table 4), and the total genetic values from the baseline model. When comparing genomic predictions to total genetic values, prediction accuracies were varied with a range between 0.55 (yellows) and 0.85 (TKW). As expected, comparing these predictions to the additive genetic values produced higher and more consistent prediction accuracies with all traits falling between 0.96 and 0.98. Using one QTL to predict performance was much less accurate with traits ranging between 0.11 (test weight) and 0.45 (glaucousness) when comparing to total genetic values, and between 0.10 (test weight) and 0.54 (greenness) when comparing to additive genetic values. The five QTL model yielded prediction accuracies ranging from 0.31 (NDVI) to 0.68 (glaucousness) when compared to total genetic values, and between 0.42 (NDVI) and 0.78 (greenness) when compared to additive genetic values. There was a strong positive relationship ($r=0.84$) between genomic prediction accuracy calculated using total genetic values and the proportion of genetic variance that was additive for the trait. This relationship was negligible for genomic prediction accuracies calculated using additive genetic values values ($r=-0.13$).

## Discussion

Previous applications of GS have predominantly used wheat germplasm collections of approximately 500 individuals (Crossa et al. 2010; Heslot et al. 2012, 2013; Dawson et al. 2013; Lado et al. 2013), while two recent studies used panels containing over 3000 individuals (He et al. 2016, 2017). This research has been invaluable in promoting the concept of GS in wheat, and providing a framework for future research. The natural progression is to work with larger datasets that provide more direct relevance to large-scale breeding programmes. In this study we used a panel of 10,375 wheat breeding lines to investigate the genomic prediction accuracy achievable in germplasm of this size and nature. We also compare these prediction accuracies to those achieved with models using a finite number of QTL, which are reflective of the style of marker-assisted selection already being used within wheat breeding programmes. We also assessed the extent of LD present in the germplasm and investigated genetic correlations between traits.

Significant LD within a training set leads to low genetic resolution and results in prediction calibrations which break down quickly in a breeding programme (Hickey et al. 2014). The panel presented here contains very low levels of LD compared to multi-parent advanced inter-cross (MAGIC) populations (Huang et al. 2012), and is more comparable to diverse germplasm collections (Chao et al. 2010; Sukumaran et al. 2015). This information, along with the high prediction accuracies we observed, highlights that our calibration successfully exploited short haplotype effects rather than long. Hickey et al. (2014) suggested that this type of calibration would retain prediction accuracy over multiple generations of inter-crossing, which future work will investigate.

The additive and residual genetic correlations between 91 trait combinations show that while the two measures commonly mirror each other, they do at times differ (glaucousness–greenness, leaf loss–maturity). A negative relationship between grain protein and grain yield has frequently been identified at a phenotypic level (Brooks et al. 1982; Jenner et al. 1991; Simmonds 1995; Oury and Godin 2007), and here we extend this understanding by showing the relationship exists at both an additive and residual genetic level. The same applies for the strong positive relationship between test weight and TKW, where phenotypic correlations were previously demonstrated by (Sharma and Anderson 2004; Rharrabti et al. 2003). Negative correlations between grain protein and test weight, as observed here, are common when plants are stressed during grain fill (Sadras et al. 2002) as the Roseworthy experiment was. The positive additive and residual genetic correlations between grain yield and relative maturity score were caused by the dry finish to the season, which favoured early maturing lines.

Incorporating the genomic relationship matrix into the linear mixed models vastly improved the model fit for all traits. This translates to more genetic variation of the trait being captured by the model, and also more accurate partitioning of variance into genetic (subsequently partitioned into additive and residual genetic) and residual error sources. The strong positive correlation between improvement in model fit and narrow sense heritability demonstrates that the additive relationship matrix improves the model by more accurately capturing additive genetic variance. Traits with a high proportion of additive genetic variance will, therefore, benefit most from the inclusion of the marker relationship matrix in the model.

The efficacy of genomic prediction is typically assessed by means of cross-validation, where predictions of the validation set are correlated to the corresponding phenotypic estimated breeding values (Crossa et al. 2010; Lado et al. 2013). These phenotypic values (in this case a best linear unbiased prediction) represent both additive and residual genetic variance, whereas the genomic prediction represents only additive genetic variance. This discrepancy between the two values results in lower perceived prediction accuracies that are skewed according to the proportion of trait variance that is additive. The results presented in Table 5 demonstrate this as the genomic prediction accuracies produced by correlating predictions to total genetic values and dividing by the square root of heritability were significantly lower than those produced by correlating to additive genetic values, and were also strongly related to the proportion of genetic variance that is additive. Correlating cross-validation predictions directly to the additive genetic values, therefore, provides a purer measure of prediction accuracy as both values contain only additive genetic variance, which prevents the proportion of additive variance from confounding the measure. Breeders can then use the prediction accuracy of a given trait (as measured by correlating to additive genetic values) to judge how effective GS will be for selecting lines with high breeding value (parents), and use both the prediction accuracy and the proportion of additive variance to judge how effective GS will be for selecting lines with high phenotypic performance (varieties). The concept of separating these two breeding objectives was investigated by Gaynor et al. (2017) and was found to significantly increase the rate of genetic gain.

Genomic prediction accuracy was very high for all traits when comparing to additive genetic values. This suggests that genomic selection is promising for all traits when the breeder is interested in additive genetic variance, i.e. when selecting parents. When assessed against total genetic values, cross-validation accuracies for grain yield, maturity, TKW, plant height and grain protein were all higher than those reported in previous studies (Crossa et al. 2010; Heffner et al. 2011b; Heslot et al. 2012, 2013; Poland et al. 2012; Dawson et al. 2013; Lado et al. 2013; He et al. 2016). The prediction accuracy improvement is likely due to larger population size of this study compared to those previous (between 254 and 2325). In addition, previous studies sometimes sourced phenotype data from multiple environments which introduce genotype by environment (GxE) variation and decrease prediction accuracy. In this study we used just one environment to remove the confounding effect of GxE and gain a more direct assessment of genomic prediction accuracy in the most optimal scenario. However, the prediction accuracies observed here were still higher than previous cross-validation accuracies produced within one environment, showing that larger population size is important in achieving high prediction accuracy.

QTL-based predictions calculated from five selected QTL were more accurate for all traits than those utilizing one QTL, while the use of genomic prediction was significantly more accurate than both. This result is in line with previous comparisons between QTL-based prediction and genomic prediction in different traits. Rutkoski et al. (2012) found that genome-wide prediction models outperformed targeted marker models for most traits related to Fusarium head blight, while Heffner et al. (2011a) showed that genomic predictions were significantly more accurate than QTL-based predictions for grain quality traits. The research presented here demonstrates that this trend holds true for grain yield, physical grain quality, and physiological traits. The traits that were most accurately predicted by QTL were greenness and glaucousness. These two traits expressed several large effect QTL (Online Resource 5) which explain their high prediction accuracy (Desta and Ortiz 2014). NDVI showed low QTL-based prediction accuracy as there were no moderate or large effect QTL influencing the trait (Online Resource 5).

The dataset used in this study represents an unprecedented resource for studying the efficacy and application of genomic selection in bread wheat. We showed that incorporating a genomic additive relationship matrix into the linear mixed model significantly improved the model fit and increased trait heritability. The fivefold cross-validation produced higher genomic prediction accuracies than those from previous studies which used smaller populations. We also showed that for all traits assessed in this research, genomic prediction was significantly more accurate than QTL-based prediction, but as expected the improvement was smaller for qualitative traits. This panel will be used in future work to investigate the effects of population size, population structure, and GxE interaction on genomic prediction accuracy.

### Addendum

Marker data will be available for downloading as supplementary material 12 months after publication, or in advance from the authors subject to the terms of a material transfer agreement.

### Author contribution statement

AN: manuscript preparation, phenotypic data generation, analysis of phenotypic and genetic data. JT: construction of the genetic linkage and consensus maps; PhD co-supervisor of Adam Norman. ET: construction of the genetic linkage and consensus maps. PT: generation of several bi-parental populations used in the genetic mapping. JE: PhD co-supervisor of Adam Norman; direction and content of research and the article. JPM: development of SNP genotyping platform. HK: PhD principal supervisor of Adam Norman; direction and content of research and the article.

## Electronic supplementary material

Below is the link to the electronic supplementary material.

Supplementary material 1 (PDF 141 kb)

Supplementary material 2 (PDF 396 kb)

Supplementary material 3 (XLS 1146 kb)

Supplementary material 4 (CSV 425000 kb)

Supplementary material 5 (XLS 26 kb)
