# Supplementary material for: Increased genomic prediction accuracy in wheat breeding using a large Australian panel
Source: Theor Appl Genet. 2017 Sep 8;130(12):2543–55. doi: 10.1007/s00122-017-2975-4 (PMC5668360; doi:10.1007/s00122-017-2975-4)
Supplement: Supplementary file 1 — Supplementary material 1 (PDF 141 kb) [file 122_2017_2975_MOESM1_ESM.pdf]

Supplementary material for:

Increased genomic prediction accuracy for wheat breeding using a large Australian panel

Adam Norman[1,3], Julian Taylor[1], Emi Tanaka[2], Paul Telfer[1,3], James Edwards[1,3], Jean-Pierre Martinant[4] and Haydn Kuchel[1,3]

[1]School of Agriculture, Food & Wine
University of Adelaide, Waite Campus
Glen Osmond, South Australia, Australia
Tel.: +61 4 0065 6012
E-mail: adam.norman@adelaide.edu.au

[2]National Institute for Applied Statistics Research Australia (NIASRA)
School of Mathematics and Applied Statistics
University of Wollongong
Wollongong, New South Wales, Australia

[3]Australian Grain Technologies Pty Ltd
Perkins Building, Roseworthy Campus
Roseworthy, South Australia, Australia

[4]Limagrain Field Seeds Pty Ltd
Centre of Research, Chappes, France

Correspondence: adam.norman@agtbreeding.com.au

Table 1 Summary of the genetic material used in the study

	# lines	Derivation	Source	Use
AWP	10375	-	-	Association panel
PYT-South	7622	F_2 - F_5	AGT	Association panel
AYT-South	865	F_5 - F_8	AGT	Association panel
AYT-Other	1888	F_5 - F_8	AGT	Association panel
Kukri / RAC875	157	DH	Bennett et al (2012)	Anchor markers & map construction
Kukri / Excalibur	132	DH	Edwards (2012)	Anchor markers & map construction
Halberd / Kennedy	121	DH	Daryl Mares (pers. comm.)	Map construction
Mace / Gladius	176	DH	(Telfer, 2013)	Map construction
Scout / Mace	226	DH	(Telfer, 2013)	Map construction
Scout / Gladius	369	DH	(Telfer, 2013)	Map construction
RAC1548 / Gladius	132	DH	(Telfer, 2013)	Map construction
AUS17750 / Gladius	116	DH	(Telfer, 2013)	Map construction
AUS17840 / Gladius	124	DH	(Telfer, 2013)	Map construction

References

Bennett D, Izanloo A, Edwards J, Kuchel H, Chalmers K, Tester M, Reynolds M, Schnurbusch T, Langridge P (2012) Identification of novel quantitative trait loci for days to ear emergence and flag leaf glaucousness in a bread wheat (*Triticum aestivum L.)* population adapted to southern Australian conditions. Theoretical and Applied Genetics 124(4):697–711

Edwards J (2012) A genetic analysis of drought related traits in hexaploid wheat. PhD thesis, The University of Adelaide

Telfer P (2013) Production of doubled haploid wheat populations for heat tolerance research, unpublished work
