# Supplementary material for: Increased genomic prediction accuracy in wheat breeding using a large Australian panel
Source: Theor Appl Genet. 2017 Sep 8;130(12):2543–55. doi: 10.1007/s00122-017-2975-4 (PMC5668360; doi:10.1007/s00122-017-2975-4)
Supplement: Supplementary file 2 — Supplementary material 2 (PDF 396 kb) [file 122_2017_2975_MOESM2_ESM.pdf]

Supplementary material for:

Increased genomic prediction accuracy for wheat breeding using a large Australian panel

Adam Norman[1,3], Julian Taylor[1], Emi Tanaka[2], Paul Telfer[1,3], James Edwards[1,3], Jean-Pierre Martinant[4] and Haydn Kuchel[1,3]

[1]School of Agriculture, Food & Wine
University of Adelaide, Waite Campus
Glen Osmond, South Australia, Australia
Tel.: +61 4 0065 6012
E-mail: adam.norman@adelaide.edu.au

[2]National Institute for Applied Statistics Research Australia (NIASRA)
School of Mathematics and Applied Statistics
University of Wollongong
Wollongong, New South Wales, Australia

[3]Australian Grain Technologies Pty Ltd
Perkins Building, Roseworthy Campus
Roseworthy, South Australia, Australia

[4]Limagrain Field Seeds Pty Ltd
Centre of Research, Chappes, France

Correspondence: adam.norman@agtbreeding.com.au

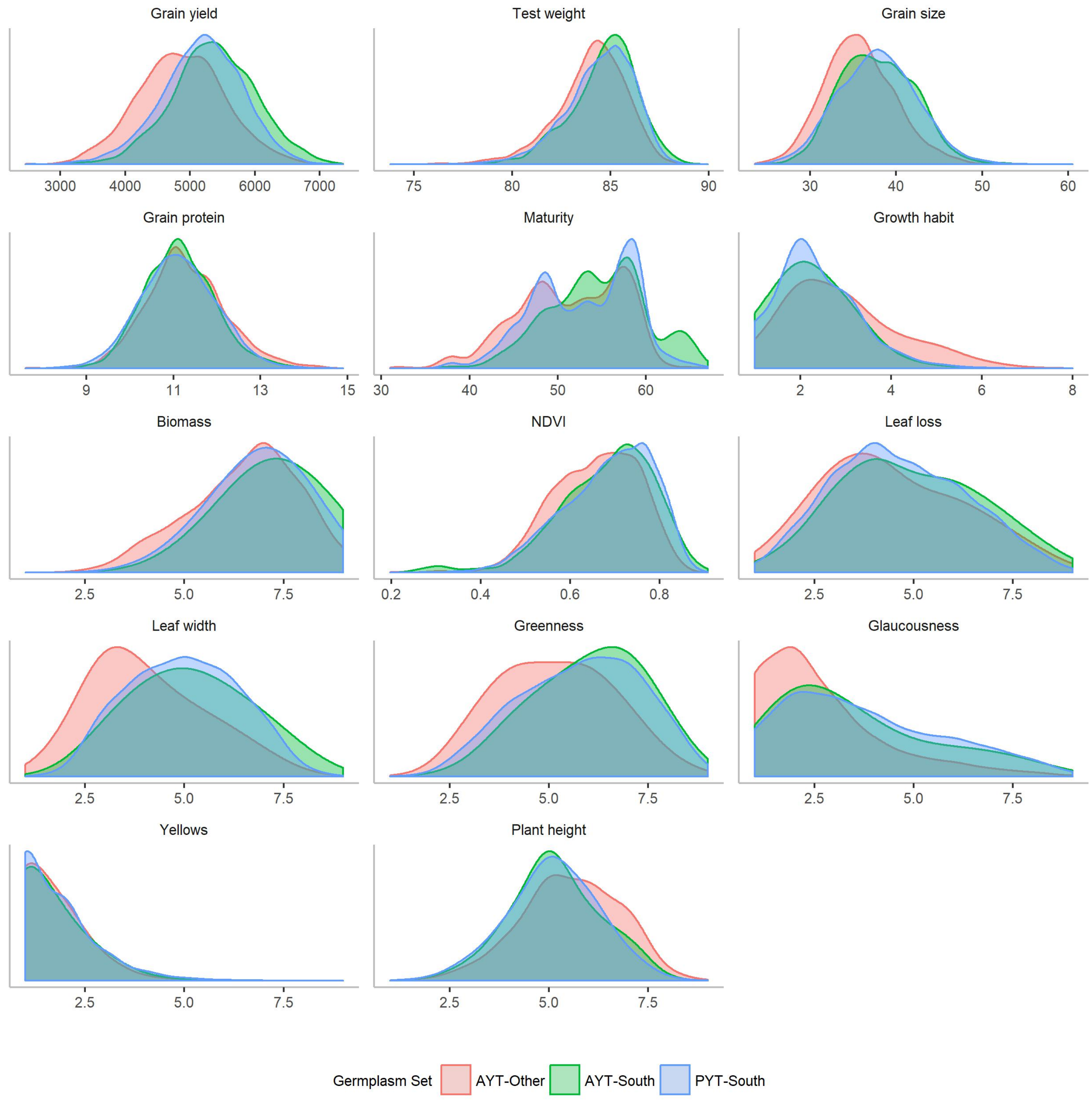
Grain yield
3000
4000
5000
6000
7000
Test weight
75
80
85
90
Grain size
30
40
50
60
Grain protein
9
11
13
15
Maturity
30
40
50
60
Growth habit
2
4
6
8
Biomass
2.5
5.0
7.5
NDVI
0.2
0.4
0.6
0.8
Leaf loss
2.5
5.0
7.5
Leaf width
2.5
5.0
7.5
Greenness
2.5
5.0
7.5
Glaucousness
2.5
5.0
7.5
Yellows
2.5
5.0
7.5
Plant height
2.5
5.0
7.5
Germplasm Set
AYT-Other
AYT-South
PYT-South
